# Sonographic changes in the thyroid gland after sclerotherapy with doxycycline can be mistaken for thyroid cancer

**DOI:** 10.1186/s13044-023-00177-1

**Published:** 2023-08-17

**Authors:** Steven Raeymaeckers, Maurizio Tosi, Bastiaan Sol, Johan De Mey

**Affiliations:** 1 Department of Radiology, Universitair ziekenhuis Brussel, Laarbeeklaan 101, 1090 Jette, Belgium; 2 Department of Anesthesiology, Universitair ziekenhuis Brussel, Laarbeeklaan 101, 1090 Jette, Belgium; 3 Department of Endocrinology, Universitair ziekenhuis Brussel, Laarbeeklaan 101, Jette, 1090 Belgium

**Keywords:** Thyroid, Thyroid malignancy, TIRADS, Sclerotherapy, Doxycycline

## Abstract

**Background:**

The literature considers sclerotherapy to be a safe and effective treatment for benign thyroid cysts. No subsequent diagnostic problems have been reported as a complication. We report the occurrence of focal inflammation after said therapy, mimicking a thyroid malignancy.

**Case presentation:**

We report a case of a young male with a solitary strongly suspicious lesion in the thyroid. The patient had undergone prior sclerotherapy of a thyroid cyst with Doxycycline. The lesion appeared to be a focal area of inflammation and thus iatrogenic in nature. Systemic use of doxycycline is known to sometimes cause a non-immune chemical thyroiditis, dubbed as black thyroid due to the intense black discoloration of the thyroid. It might be that the instillation of doxycycline was responsible for a similar, more localized area of thyroiditis.

**Conclusions:**

For the work-up of a solitary suspicious thyroid lesion, the medical history of the patient should always be considered. In case of prior ipsilateral sclerotherapy, a reactive inflammatory response may mimic thyroid malignancy. A fine needle aspiration should be performed to exclude thyroid cancer. Treatment is not necessary; the process appears to be self-limiting as evidenced in the follow-up of this case.

## Background

In case of benign thyroid cysts, percutaneous tetracycline instillation, ethanol sclerotherapy, or thyroid hormone suppression therapy can be performed [1]. Thyroid hormone suppression therapy is no longer advocated, as the effect of medication in volume reduction of a thyroid nodule is uncertain and side-effects are to be expected [2]. Several sclerosants stimulate fibrous union of the tissue surfaces by inducing inflammation [3]. In our institution we opted to use doxycycline as a sclerosing agent, as it proved in our experience to be an effective and safe sclerosant for the treatment of seromas [3].

Doxycycline is an inexpensive drug and used to be readily available in its parenteral form. It is an antibiotic of the tetracycline family and its safety has been well established [4, 5]. Apart from its antibiotic properties, Doxycycline can be used as a sclerosant, for instance to treat pleurodesis of malignant effusions [6] or as treatment for lymphoceles [7] and lymphatic malformations [8, 9]. Parenteral forms of the drug are not available anymore in a number of countries (including the USA). It has since been established that treatment with a saline solution of the oral form of the drug is equally effective [10].

## Case presentation

A 39-year-old male patient presented for routine follow up at our endocrine clinic. He had previously been treated for a recurrent bleeding cyst in the thyroid with sclerotherapy using doxycycline. Treatment had been performed because of progressive swallowing difficulties experienced by the patient, who refused surgical options. Initial aspiration of the cyst was inconclusive (Bethesda 1) but because of recurrence of the cyst, sclerotherapy was performed. One year after treatment, a mildly painful swelling of the right thyroid lobe was noted by the endocrinologist upon physical examination. No other abnormalities were present. Thyroid-stimulating hormone level was measured at 1.26 mIU/L (normal range 0.4 to 4.0 mIU/L), free T4 at 1.6 ng/dL (normal range 0.8 to 1.8 ng/dL). Free T3 was discretely elevated, measuring 4.3 pg/L (normal range 2.3–4.1 pg/mL).

**Fig. 1:**
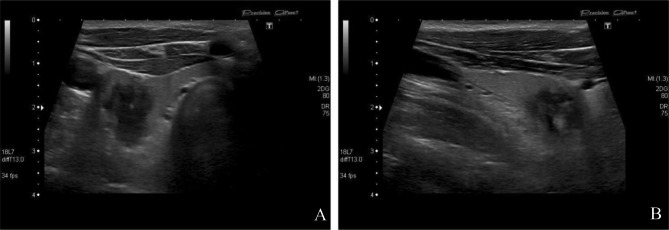
Ultrasound of the thyroid gland A (left): Axial plane. B (right) sagittal plane. Lesion in the lower part of the thyroid. The lesion is solid, hypoechoic with irregular borders. The lesion is also taller than wide and seems to contain some microcalcifications

Ultrasound revealed a large suspicious lesion in the right thyroid lobe. This lesion is illustrated in Fig. 1. The lesion appeared solid and markedly hypoechoic with irregular borders. It measured 14 mm in craniocaudal length. In the axial plane it measured 15 mm in anteroposterior lengrth as opposed to 9 mm in laterolateral length, making this lesion taller than wide. It alsoseemed to contain some microcalcifications. Based on these criteria the lesion was graded as a TIRADS 5 lesion, necessitating a fine needle aspiration.

**Fig. 2:**
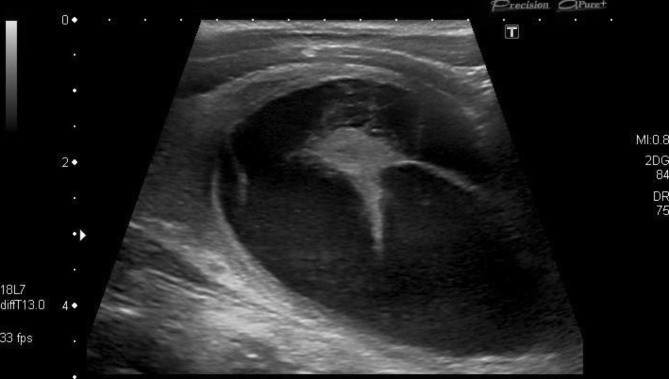
Ultrasound of the thyroid gland Ultrasound prior to sclerotherapy, one year earlier. Note the large bleeding cyst in the right thyroid lobe

Upon examining the patient’s medical history, we found evidence of the aforementioned sclerotherapy with Doxycycline. The patient had undergone this procedure one year earlier to treat a benign thyroid cyst in the right thyroid lobe (Fig. 2). Upon examination of the prior ultrasound images, this cyst (although more voluminous in nature) appeared to be localized in the same region as the suspicious lesion.

**Fig. 3:**
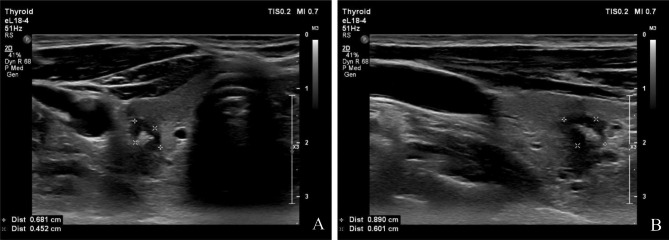
Ultrasound of the thyroid gland A (left): Axial plane. B (right) sagittal plane. Check-up ultrasound, 4 years later. The lesion is clearly smaller in size and with a different morphology: progressive formation of a macrocalcification and now smooth borders

Regardless, we decided to perform a fine needle aspiration to exclude an underlying thyroid malignancy. The fine needle aspiration sample was qualitative for diagnosis. The sample showed a relatively prominent amount of aqueous to slightly thickened colloid with some groups of follicle cells, as well as the presence of neutrophils, lymphocytes, and histiocytes. The sample was graded as a Bethesda 2 result. In this light, we considered the inflammation to be reactive following sclerotherapy and decided to follow up with ultrasound. Follow up ultrasound examinations showed no enlargement of the suspicious lesion, over the course of the next 4 years the lesion would even gradually reduce in size. In Fig. 3 we illustrate the follow-up ultrasound 4 years after the initial imaging, i.e. 5 years after sclerotherapy. On these images the lesion is found to be clearly smaller in size with a different morphology: the progressive formation of a macrocalcification and smooth borders of the lesion now more resemble a benign colloid nodule.

## Discussion and conclusions

Ultrasound is the imaging modality of choice for the evaluation of the thyroid and the risk stratification/follow up of thyroid nodules. Many different proposals of a Thyroid Imaging Reporting and Data System (TIRADS) have been described in the literature. For the description of thyroid nodules in our hospital, we adhere to the system as proposed by Kwak et al. [11] Regardless of the TIRADS system used, several described sonographic features are nearly universal. Lesions that are mainly solid, hypoechoic or even markedly hypoechoic are more suspicious than lesions that are mixed or predominantly cystic. Lesion contours, taller-than-wide aspect and microcalcifications are other remaining factors that should be considered.

The literature considers sclerotherapy to be a safe and effective treatment for benign thyroid cysts. Sclerotherapy is almost exclusively performed by percutaneous ethanol injection (PEI), the safety of this procedure has been well established since the introduction of the technique in 1990 by Livraghi et al. [12] These authors followed 101 patients over the course of 4 years to evaluate the long-term efficacy of their proposed treatment.

A large clinical trial on the use of PEI was subsequently conducted by Lee et al. [13] and included 654 patients. Not only did these authors opt to treat thyroid cysts, but they treated solid nodules as well. Complete response, partial response and no response were found to be 17.2%, 71.7%, 11.1% in solid nodules as compared to 19.0%, 60.4%, 20.6% for complex cysts, respectively.

Minimally invasive treatment of symptomatic thyroid nodules has since become commonplace. Therefore, the Task Force Committee of the Korean Society of Thyroid Radiology initiated a consensus statement providing recommendations for the role of PEI in the management of symptomatic thyroid nodules [14]. These guidelines consider different known complications of sclerotherapy, such as localized pain, hematoma, facial flushing, drunken sense, hoarseness, dyspnea, and temporary hyperthyroidism. In malignant cases, tumor implantation can occur as a very rare complication that can occur months to years after PEI [15].

The patient described in this report was also treated for a benign thyroid cyst, with doxycycline as a sclerosing agent. None of the aforementioned complications was present in our case, however a routine follow-up one year later found a large suspicious lesion present in the treated region of the thyroid. Considering the timeframe, the localization of this lesion and the inflammatory cells found in the fine needle aspirate, it seems logical to consider a link to the prior treatment. No other case can be found in the literature in which a diagnostic problem arose after PEI treatment of a thyroid cyst, nor in those few studies where different sclerosing agents were used.

It could be we witnessed a common but underreported effect of thyroid sclerotherapy in general, due to small patient groups and lack of follow-up. If this is the case, patients who have undergone sclerotherapy might be at risk of false-positively being diagnosed with thyroid malignancy if the relevant medical history is not considered. The aforementioned study by Lee et al. [13] however included 654 patients who were followed up for 36 months after PEI treatment and did not report on any suchcomplications.

As sclerotherapy is usually performed with ethanol, it can then be suggested that doxycycline is specifically the agent to blame in this case. Systemic use of doxycycline is known to sometimes cause a non-immune chemical thyroiditis [16], dubbed as black thyroid due to the intense black discoloration of the thyroid [17]. It might be that the instillation of doxycycline was responsible for a similar, more localizedarea of thyroiditis.

In conclusion we would suggest that for the work-up of a solitary suspicious thyroid lesion, the medical history of the patient should always be considered. In case of antecedents of ipsilateral sclerotherapy, a reactive inflammatory response may mimic thyroid malignancy. In this specific case Doxycycline was responsible, an effect that may specifically be explained by the occurrence of non-immune chemical thyroiditis. It is not known if other sclerosants may elicit the same effect, however this complication was not reported in large follow-up studies that use ethanol as sclerosing agent. It could however be that these inflammatory changes post sclerotherapy have not been reported as this may be thought of by different authors as a normal process. Fine needle aspiration should be performed to exclude thyroid cancer. Treatment is not necessary; the affliction appears self-limiting as evidenced in the follow-up of this case.

## Data Availability

Not applicable.

## List of abbreviations

TIRADS: Thyroid Imaging Reporting and Data System
PEI: Percutaneous ethanol injection
